# Optimization of oil-in-water emulsion capacity and stability of octenyl succinic anhydride-modified porang glucomannan (*Amorphophallus muelleri* Blume)

**DOI:** 10.1016/j.heliyon.2022.e09523

**Published:** 2022-05-24

**Authors:** I Wayan Rai Widarta, Ambar Rukmini, Umar Santoso, Supriyadi, Sri Raharjo

**Affiliations:** a Department of Food Technology, Faculty of Agricultural Technology, Udayana University, Bali, Indonesia; b Study Program of Food Technology, Faculty of Science and Technology, Widya Mataram University, Yogyakarta, Indonesia; c Department of Food and Agricultural Product Technology, Faculty of Agricultural Technology, Universitas Gadjah Mada, Yogyakarta, Indonesia

**Keywords:** Surfactant, Porang glucomannan, Emulsion, Sodium carbonate, Octenyl succinic anhydride

## Abstract

Surfactants are used to reduce surface and interfacial tension to form emulsions. Polysaccharides such as Porang Glucomannan (PG) with high viscosity can be used as surfactants. This research aimed to optimize the concentration of sodium carbonate (Na_2_CO_3_) and octenyl succinic anhydride (OSA) in modifying PG using a microwave. The optimization process is carried out using response surface methodology (RSM) with a two-factor central composite design (CCD), namely concentration of Na_2_CO_3_ (0.17–5.834%) and OSA (2.17–7.83%). The result showed that the concentration of Na_2_CO_3_ and OSA strongly influences emulsion capacity and stability. The optimum conditions that resulted in the highest emulsion capacity and stability were obtained at concentrations of Na_2_CO_3_ and OSA which were 2.25% and 6.19%, respectively. Degree of Substitution (DS), FTIR analysis, contact angle, and increased viscosity confirmed that OSA substitution occurred in PG. The characteristics of OSA-modified porang glucomannan (PGOS) such as: emulsion capacity and stability, Degree of Substitution (DS), contact angle, and viscosity increased to 34.6% and 32.5%, 1.02%, 92^o^, 5720 cP, respectively. FT-IR analysis confirmed the presence of OSA substitution at 1734 cm^−1^. PGOS can be used as a surfactant or gelator in oleogel production.

## 1 Introduction

Surfactants are defined as 'surface active agents' which are amphiphilic substances because they contain hydrophilic (polar) and hydrophobic (nonpolar) groups. Surfactants reduce surface and interfacial tension to form micelles/emulsions. Surfactants are widely applied in the food and non-food industries (Sun et al., 2022). Meng et al. (2014) reported that polymer surfactants have great potential to be developed because their raw materials are diverse, flexible in their use with higher functionality values.

Glucomannan is a non-ionic linear polysaccharide. The main chain of glucomannan consists of D-mannose and D-glucose with a α-1,4-pyranoside bond and acetyl groups substitution. The ratio of mannose to glucose is 1.6:1. There is a branching point in one of the leading chains, C-3 hydroxyl groups of glucose or mannose, with a branched-chain length of 3–4 monosaccharides (Wang et al., 2015). The main chain of konjac glucomannan (KGM) is partially acetylated (5–10%) at the C-6 position, which causes konjac glucomannan to be water-soluble (Zhong et al., 2018; Zhang et al., 2014). This acetyl strengthens hydrogen bonds with water and reduces hydrophobic interactions (Wardhani et al., 2017). Glucomannan is applied in the food sector as a food additive because it has properties as a thickener, and glucomannan can form a good gel. KGM is also used in producing low-fat cheese, as a fat substitute in yogurt and fat analogs in mayonnaise, and as a stabilizer in ice cream.

Glucomannan can also be obtained from Porang tubers. Porang (*Amorphophallus muelleri* Blume) is widely found in tropical forests in Indonesia. Porang is also widely cultivated in Indonesia. Indonesian porang was exported to several countries, especially China, Vietnam and Thailand with a volume reaching 11,170 tons in 2019 (Dermoredjo et al., 2021). Porang has a high glucomannan content. The yield of glucomannan isolated directly from fresh porang tubers reached 50.0%–65.2%, with a purity of 77–91% depending on the method used (Yanuriati et al., 2017). Glucomannan is one of the gelling components widely applied in the manufacture of hydrogels (Yang et al., 2017; Ye et al., 2019).

Glucomannan can form good film and gelation, is environmentally friendly, and is biocompatible. Glucomannan has been approved as a food additive in Europe, and it is classified as GRAS (Generally Recognized as Safe) by the Food and Drug Administration (FDA) (Yang et al., 2017). For porang glucomannan to be used as a surfactant, it is necessary to modify glucomannan to have amphiphilic properties. Modification using octenyl succinic anhydride (OSA) produce starch with amphiphilic and surface-active properties by involving the partial substitution of starch hydroxyl groups with hydrophobic octenyl groups and the carboxyl or sodium carboxylate group (Altuna et al., 2018).

Meng et al. (2018) reported the glucomannan modification process in konjac tubers by esterification using OSA to obtain its hydrophobic properties. KGM esterified with OSA (KGOS) has better emulsifying and thickening ability than native konjac glucomannan flour and commercial OSA esterified starch (OSAS), so it has the potential to be used as a polymer surfactant in the industry (Meng et al., 2014). In addition, according to the FDA, OSAS is approved as GRAS for food with a maximum limit of 3% OSA per starch weight (No et al., 2019). Zhao et al. (2018) reported that succinylation replaces the hydroxyl group in starch molecules with OSA carbonyl groups. Partial substitution of hydroxyl groups with hydrophobic compounds resulted in amphiphilic character in OSAS so that it can be used as an emulsifier and encapsulant for hydrophobic bioactive.

Factors that affect the modification of glucomannan are the concentration of Na_2_CO_3_ and OSA. Modifying KGM with OSA in a slightly alkaline solution has caused deacetylation of some of the acetyl groups of KGM because OSA can inhibit the deacetylation reaction. Wardhani et al. (2017) reported that the addition of Na_2_CO_3_ resulted in a deacetylation reaction in glucomannan. The increased concentration of Na_2_CO_3_ in the glucomannan deacetylation process resulted in decreased solubility caused by the loss of acetyl groups during deacetylation. During deacetylation, the steric hindrance is removed so that the polymer is easier to associate through hydrophobic interactions which causes the gelling ability to increase. Meng et al. (2018) reported that the addition of carbonyl groups and deacetylation occurred simultaneously during the modification process of KGM with OSA. The increase of the degree of substitution (DS) up to 1.51% increased the emulsion capacity and emulsion stability of KGOS (Meng et al., 2014).

Modifications can be carried out using a microwave quickly, as in the modification of OSA starch. Modifying starch with OSA using a microwave has produced OSA starch with high DS quickly; for example, DS was 0.021 in 7 min (Mahajan and Sonar, 2019). The interaction of components in solution is faster due to the presence of molecular friction and excitation. This interaction results in more efficient internal heating (Manab et al., 2016). Mahajan and Sonar (2019) reported that modification of pea starch with OSA using microwave irradiation could increase its emulsifying ability and oil retention ability. This study aimed to find the optimum conditions (Na_2_CO_3_ and OSA concentration) to obtain OSA esterified glucomannan porang (PGOS), which has amphiphilic properties. Maximum emulsion capacity and stability were used as indicators of the success of the modification process.

## 2 Materials and methods

### 2.1 Materials

PG (Porang glucomannan) with 99.4% purity was purchased from a local producer, extracted and refined from the dried chip of Porang (*Amorphophallus muelleri* Blume) tuber. The 2-Octen-1-yl succinic anhydride (OSA, 99.0% purity), ethanol absolute, and sodium carbonate were purchased from Sigma Chemical Co. (St. Louis, MO), and hydrochloric acid 37% was purchased from Mallinckrodt Baker, Inc.

### 2.2 Methods

#### 2.2.1 Preparation of PGOS

The microwave method is used to prepare PGOS (Meng et al., 2014). Ten grams of PG (dry weight) were added into a 100 mL glass bottle with a lid. Na_2_CO_3_ and OSA with a certain concentration were added (percentage based on PG, w/w). Previously, OSA was dissolved five times with absolute ethanol (v/v). Next, 40 mL of 30% ethanol was added and agitated slowly for 5 min. The solution is then placed in an Electrolux microwave reactor (Stockholm, Sweden) and heated at 800 W for 3 min. After that, the solution was cooled to room temperature, and 40 ml of 30% ethanol was added. HCl solution (1N) was used to adjust the pH of the solution to 6.50. The solution was then centrifuged at 3000 rpm for 10 min. The supernatant was discarded, while the residue was washed five times with 30% ethanol and absolute ethanol, respectively. Subsequently, the solids were dried in a cabinet dryer at 50 °C for 3 h. After drying, the PGOS was ground using a mortar and sieved through a 100-mesh nylon sieve.

#### 2.2.2 Viscosity, emulsion capacity (EC), and emulsion stability (ES) tests

One percent of PGOS and PG solutions were heated at 60 °C for 20 min and then cooled to room temperature. The apparent viscosity of the sample was measured by Brookfield (DV2T, US) viscometer at 30 ± 0.5 °C at 30 rpmin with spindle #64.

According to Meng et al. (2018), the EC and ES were tested: PGOS samples were weighed as much as 1% based on the weight of the corn oil (0.1 g) dissolved in 40 ml of distilled water. The solution was then heated to 60 °C for 20 min. After that, the solution was cooled to room temperature, and 10 g of corn oil was slowly added while homogenizing with an Ultra-turax homogenizer (T50 Basic, IKA-Werke, Germany) at 6000 rpm for 1 min. The emulsion was then poured into a 50 ml centrifuge tube and centrifuged (DM0636 Multi-purpose centrifuge, DLAB, US) for 10 min at 3000 rpm. The EC and ES were calculated by Eqs. (1) and (2), respectively:

(1) EC = (height of emulsion layer/total height) x 100%

The sample was then stored at room temperature for 30 days, then used for the determination of ES.

(2) ES = (height of remaining emulsion layer/total height) x 100%

#### 2.2.3 Degree of substitution (DS)

High-performance liquid chromatography (HPLC) method was used to determine DS in PGOS (Zhong et al., 2018). An LC-20A HPLC (Shimadzu, Japan) with a Thermo BDS C18 column (Shim-Pack GIST, 5 μm C18; 4.6 × 150 mm, Shimadzu, Japan) was used to determine DS and a UV detector (Shimadzu, Japan) operated at 200 nm. As the mobile phase, a solution of acetonitrile and water with 0.1% TFA (55:45, v/v) was used with a flow rate was 1.0 mL/min by isocratic mode for 10 min. Standard curves for OSA, Free and bound OSA, and DS were determined using HPLC as Zhong et al. (2018) described.

A standard curve of OSA was prepared by weighing 0.1052 g OSA reagent added to a 25-mL volumetric flask then dissolved with acetonitrile to the desired volume. The OSA solution was pipetted into a 3 mL beaker and added 5 mL of 1 M NaOH in ethanol solution (30%). The sample was covered with plastic wrap and stirred at 400 rpm for 12 h. Next, 1.5 ml of 4 M HCl in ethanol solution (30%) was added and poured into a 10-mL volumetric flask, then diluted with acetonitrile to the desired volume. OSA standards of 1.5; 3; 6; 9; 12; or 15% was prepared by dissolving the sample with acetonitrile.

Determination of free OSA content of PGOS was carried out by as much as 0.1 g of PGOS (dry weight) was weighed in a beaker, then 5 mL of methanol was added and stirred for 1 h. The solution was centrifuged at 400 rpm, 25 °C, for 15 min. The supernatant (0.5 mL) was pipetted and dissolved with 5 mL of 1 M NaOH in ethanol solution (30%) then stirred at 400 rpm for 12 h. Next, 1.5 mL of 4 M HCl in ethanol solution (30%) was added and cooled to room temperature. Next, 3 mL of the sample was dissolved with acetonitrile up to 10 ml in a volumetric flask. The residue was removed with a 0.45 μm membrane filter. The OSA content (W_free_, g) was calculated using the total peak area (A_free_) and standard curves.

Total OSA content was determined by weighing PGOS (0.1 g dry weight) in a beaker, added 5 mL of 1 M NaOH in ethanol solution (30%), then stirred with a magnetic stirrer at 400 rpm for 12 h. Then, 1.5 mL of 4 M HCl in ethanol solution (30%) was pipetted, and put into a beaker and cooled to room temperature. The sample was pipetted (3 mL) and then diluted with acetonitrile up to 10 mL in the volumetric flask. The residue was removed with a 0.45 μm membrane filter. OSA solutions were analyzed by HPLC to obtain the total peak area (A_total_) and related weight (W_total_, g) using a standard curve. The degree of substitution (DS) is calculated by Eq. (3):

(3) $$\text{DS}=\frac{\text{OSAtotal}-\text{OSAfree}}{\text{m}}\text{x}100\text{\%}$$

Where m is the dry weight of PGOS.

#### 2.2.4 Hydrophobicity

The main parameter of hydrophobicity of a material is the static contact angle, which is defined as the angle formed between a liquid and a solid. A contact angle meter (DSA25, Krüss Co., Ltd., Germany) is used to measure the water contact angle equipped with a CCD camera and image analysis software. PGOS solution with a concentration of 1% (w/v) in 25 mL of distilled water is heated at 60 °C for 20 min. The PGOS solution was placed in a plastic mold with a diameter of 8 cm and dried in a cabinet dryer for 24 h to obtain a film. Furthermore, the contact angle was measured by dripping 3.0 μL of water on the film΄s surface (2.0 cm × 2.0 cm) and then recorded with the camera after 45 s (Li et al., 2019).

#### 2.2.5 Fourier transform infrared (FT-IR) spectroscopy

An FT-IR spectrometer (Spectrum 100; Thermo Scientific Nicolet iS10, USA) was used to qualitatively analyze PG and PGOS in a wavenumber range of 400–4000 cm^−1^ with a resolution of 8 cm^−1^ (Meng et al., 2018). PG and PGOS mixed with KBr (1:50), then dried at 105 °C for 12 h and pressed into tablets for analysis. All spectra were scanned 32 times with background and ATR correction.

#### 2.2.6 Experimental design

The effect of the independent variable (Na_2_CO_3_ concentration, X1; OSA concentration, X2) on emulsion capacity and stability was estimated using Response Surface Methodology (RSM). The independent variables and their levels used in the response surface design can be seen in Table 1. A Central Composite Design (CCD) is used to design experiments with five central point replicates was employed for the study. The CCD matrix can be seen in Table 2. Each treatment code was repeated three times. The data obtained were analyzed with Minitab 19 to estimate the independent variable's response and graph the contour plot and response surface. An empirical model describes response correlation to the modification process conditions. Model quadratic equation based on second-order quadratic for the two independent variables can be described as Eq. (4):

(4) $$Y=\beta_{0}+\sum_{i=1}^{k}\beta_{i}X_{i}+\sum_{i=1}^{k}\beta_{ii}X_{i}^{2}+\sum_{i=1,j=2}^{k-1,k}\beta_{ij}X_{i}X_{j}+\varepsilon$$

Where Y is the response variable; β_0_ is the intercept; β_i_, β_ii_, and β_ij_ are coefficients of the linear effect, double interactions; x_i_, x_j_ are the independent variables or factors and ε is an error (Sadhukhan et al., 2016).

**Table 1:** Independent variables and their levels used in the response surface design.

Independent variables	Factor level				
	-1,414	-1	0	1	1,414
Concentration of Na_2_CO_3_ (%) (X1)	0.17	1	3	5	5.83
Concentration of OSA (%) (X2)	2.17	3	5	7	7.83

**Table 2:** The CCD matrix.

Treatment No.	Code		Concentration (%)	
	X1	X2	X1	X2
1	-1	-1	1	3
2	1	-1	5	3
3	-1	1	1	7
4	1	1	5	7
5	-1.414	0	0.17	5
6	1.414	0	5.83	5
7	0	-1.414	3	2.17
8	0	1.414	3	7.83
9	0	0	3	5
10	0	0	3	5
11	0	0	3	5
12	0	0	3	5
13	0	0	3	5

#### 2.2.7 Validation step

The optimum conditions that produce the highest emulsion capacity and stability based on the estimated response were then validated to ensure the actual value. Determination of optimal conditions at the validation stage was carried out using the response optimizer on Minitab 19. At this stage, PGOS characterization was also carried out including emulsion capacity and stability, degree of substitution, hydrophobicity (contact angle), viscosity, and FT-IR analysis.

## 3 Results and discussion

### 3.1 Influence of modification conditions on emulsion capacity

Response surface plots were constructed by plotting the result from emulsion capacity against Na_2_CO_3_ and OSA concentrations to determine the optimum concentration of both factors. Based on estimated regression coefficients and Analysis of Variance (ANOVA) (Table 3), emulsion capacity was significantly influenced by the concentration of Na_2_CO_3_ and OSA.

**Table 3:** Estimated regression coefficients and ANOVA for emulsion capacity (EC) and emulsion stability (ES).

Term	Estimated regression coefficient			
	EC	P-value	ES	P-value
Intercep βo	11.70		-1.92	
Model		0.000		0.000
Linear		0.000		0.000
X1	3.25	0.001	6.35	0.000
X2	6.05	0.001	8.99	0.004
Square		0.000		0.000
X1∗X1	-0.880	0.001	-1.836	0.000
X2∗X2	-0.469	0.020	-0.819	0.011
Interaction				
X1X2	0.061	0.774	0.363	0.288
Lack of fit		0.075		0.084
R^2^	93.59%		94.67%	
R^2^adj	89.02%		90.86%	

**Figure 1:**
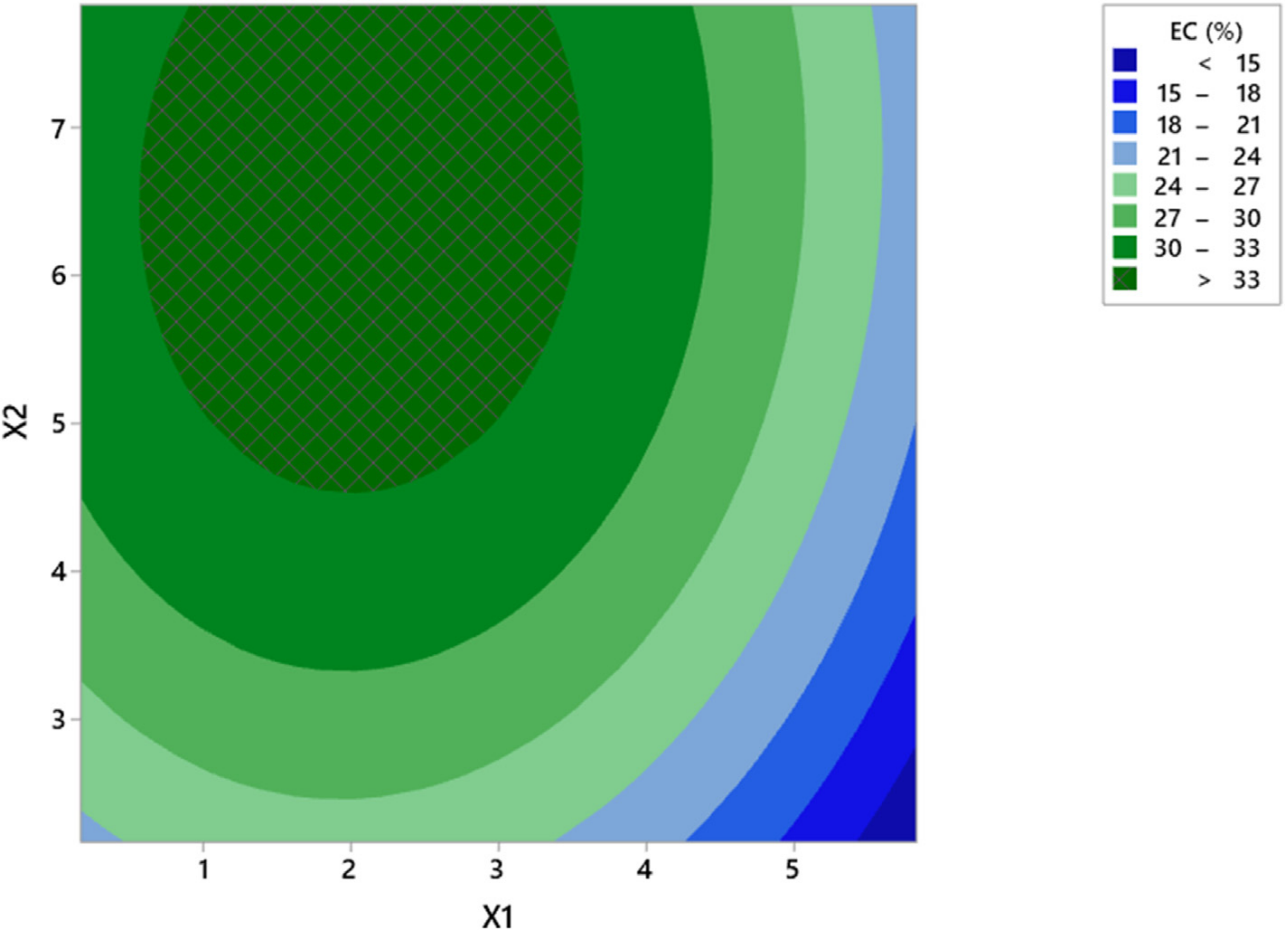
Contour plot of the effect of Na_2_CO_3_ and OSA concentration on emulsion capacity.

Table 3 shows that linear and quadratic coefficients had significant influence (*p* < 0.05), while the interaction coefficient did not (*p* > 0.05). . The coefficient of determination (R^2^) is used as information about the model fitting. Table 3 shows that the R^2^ value is 93.6%, indicating the model is well fitted and that 93.6% of the variable variance is dependent on Na_2_CO_3_ and OSA concentrations. The value of adjusted determination coefficient (R^2^adj = 89.0%) being close to R^2^ confirms high significance of the deduced model. The suitability of the model can also be investigated through a lack-of-fit test. A non-significant lack-of-fit value (*p* > 0.05) indicates the presence of the suitability of the response data with the model (Soto-Muñoz et al., 2021). Table 3 shows that the *p*-value for the lack-of-fit emulsion capacity is 0.075 (*p* > 0.05), which indicates a non-significant lack-of-fit. The 3-D response surface plot and the contour plot in Figure 1 show the emulsion capacity as a function of Na_2_CO_3_ (X1) and OSA concentration (X2). Higher concentrations of Na_2_CO_3_ and OSA resulted in increased emulsion capacity, but at higher concentrations, the emulsion capacity decreased. The addition of Na_2_CO_3_ causes deacetylation of glucomannan and simultaneously esterification of the OSA group occurs. However, at higher OSA concentrations it can inhibit the deacetylation reaction.

An alkalizing agent is needed to improve the efficiency of the polysaccharide esterification reaction. Slightly alkaline conditions can reduce hydrogen bonds in the starch chain and improve the esterification of OSA molecules in starch (Mahajan and Sonar, 2019). Na_2_CO_3_ can be used as an alkalization agent because ittriggers deacetylation reaction in glucomannan. The increasing amount of alkali causes an increase in the degree of deacetylation (Qiao et al., 2022). Li et al. (2014) also reported that adding a higher amount of alkali could increase the deacetylation reaction, but it is challenging to remove all the acetyl unless it is added in excess. However, the presence of OSA inhibited the deacetylation reaction (Meng et al., 2014). Meng et al. (2018) reported that esterification and deacetylation reactions co-occur (Meng et al., 2018). Increasing the amount of Na_2_CO_3_ and OSA up to a certain amount can increase the esterification reaction between glucomannan and OSA molecules. A higher amount of OSA is required to increase the degree of substitution (Zainal Abiddin et al., 2015).

### 3.2 Influence of modification conditions on emulsion stability

**Figure 2:**
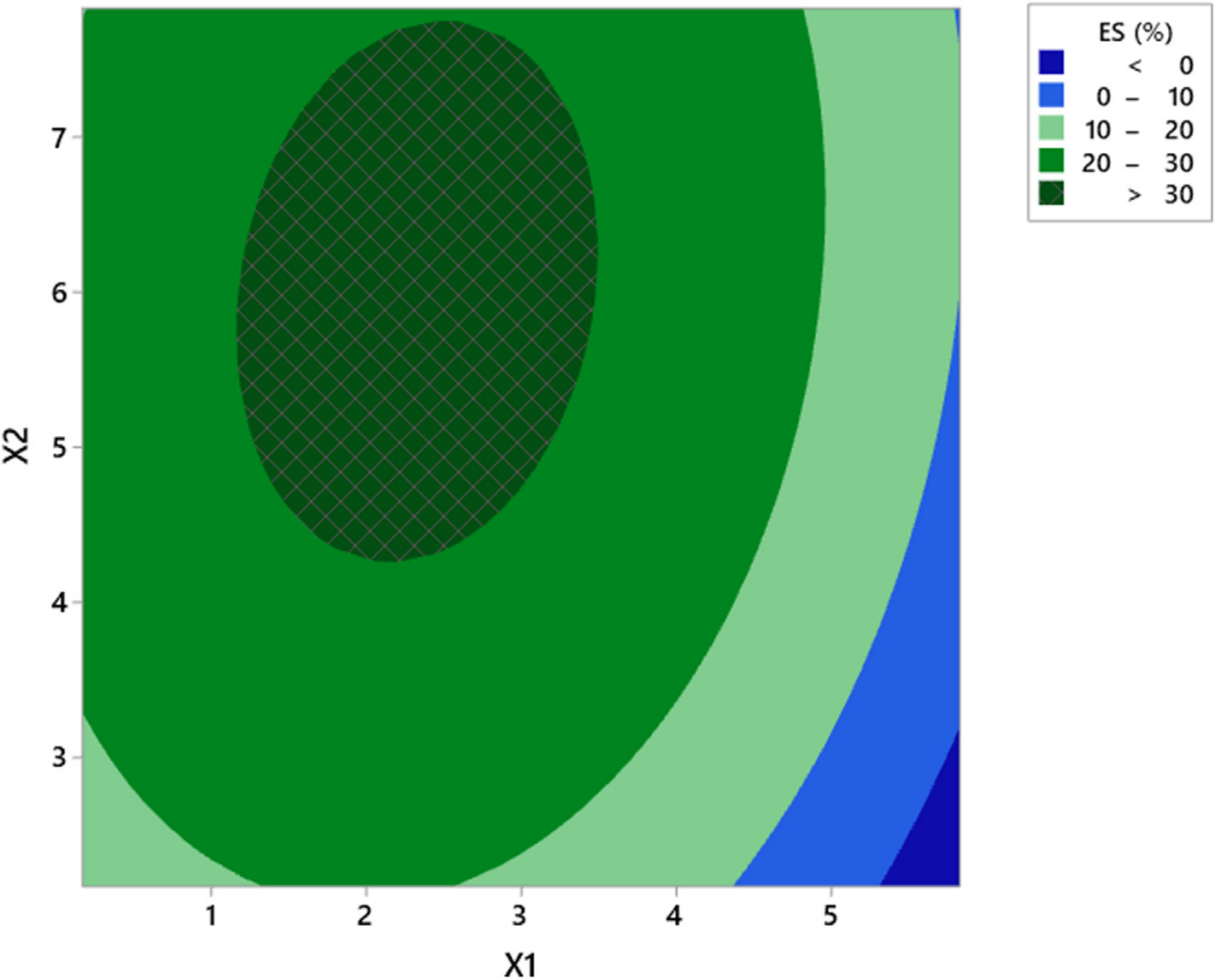
Contour plot of the effect of Na_2_CO_3_ and OSA concentration on emulsion stability (ES).

Based on estimated regression coefficients and ANOVA (Table 3), emulsion stability is strongly influenced by the concentration of Na_2_CO_3_ and OSA. The ANOVA showed that linear and quadratic coefficients were significant (*p* < 0.05), but the interaction coefficient was non-significant (*p* > 0.05). The coefficient of determination (R^2^) was 94.7%, indicating that the model is well fitted and that 94.7% of the variable variance is dependent on Na_2_CO_3_ and OSA concentrations. The value of adjusted determination coefficient (R^2^adj = 90.9%) being close to R^2^ confirms high significance of the deduced model. Table 3 shows that the *p*-value for the lack-of-fit emulsion stability is 0.084 (*p* > 0.05), which indicates a non-significant lack-of-fit. The 3-D response surface and contour plots in Figure 2 show the emulsion stability as a function of Na_2_CO_3_ (X1) and OSA concentration (X2).

The effect of Na_2_CO_3_ and OSA concentrations showed a curvature (quadratic) response where the emulsion capacity and stability increased to a maximum point and then decreased with increasing concentrations of Na_2_CO_3_ and OSA. The increase in OSA concentration increases the esterification between glucomannan and OSA. Therefore, the emulsion capacity and stability also increased (Zainal Abiddin et al., 2015). However, an excessive amount of OSA can inhibit the deacetylation reaction of Na_2_CO_3_, thus, resulting in a decrease in the OSA substitution in porang glucomannan (Meng et al., 2014). Shi et al. (2016) reported that increasing OSA can produce stable emulsions because it can reduce the droplet size of the emulsion. In addition, the increase in viscosity after OSA modification can improve emulsion stability by inhibiting the free movement of oil droplets, thereby reducing the occurrence of creaming, flocculation, and coalescence (Xu et al., 2015).

### 3.3 Validation step

The validation stage is carried out to verify the validity of the estimated value obtained based on the RSM with the actual value. Determination of optimal conditions at the validation stage is carried out using the response optimizer on Minitab 19.

#### 3.3.1 Emulsion capacity and stability

**Figure 3:**
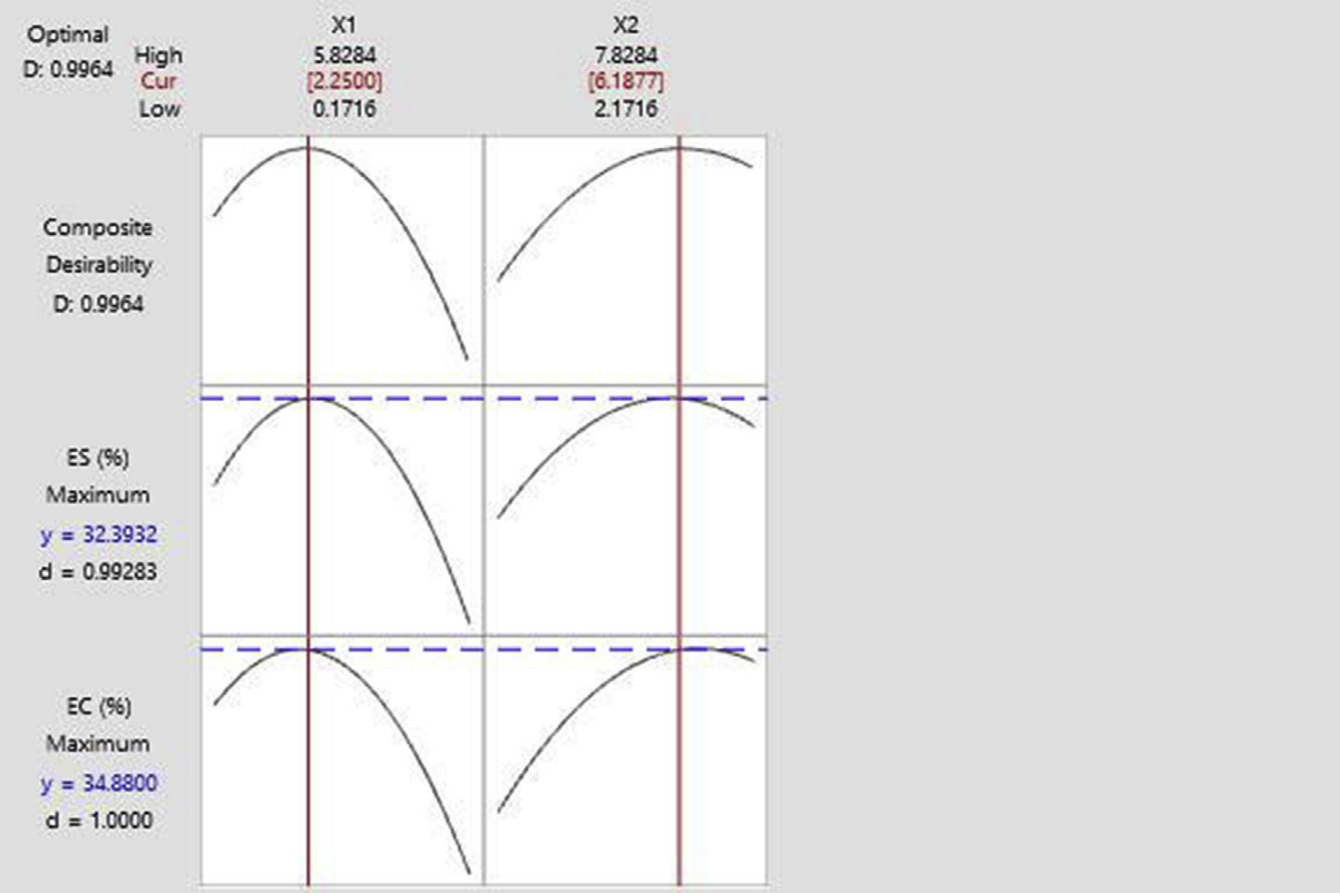
Na_2_CO_3_ (X1) dan OSA (X2) optimum concentration based on emulsion capacity and emulsion stability.

Figure 3 shows the optimum concentration of Na_2_CO_3_ (X1) and OSA (X2) to achieve the greater results of emulsion capacity and emulsion stability. The addition of Na_2_CO_3_ and OSA at concentrations of 2.25% and 6.19%, respectively will produce a maximum emulsion capacity and stability of 34.88% and 32.39%, respectively. The desirability value that is closer to 1 indicates a more desirable and credible optimal condition (Soto-Muñoz et al., 2021).

Table 4 shows the characteristics of PG and PGOS obtained at optimum conditions. T-test independent sample showed a significant difference in the characteristics of PG and PGOS (*sig.* (*2-tailed*) < 0.05). The value of PGOS emulsion capacity and stability obtained is very close to the predicted value of 34.6% and 32.5, respectively.

**Table 4:** Characteristic of porang glucomannan (PG) and octenyl succinic anhydride modified porang glucomannan (PGOS).

Characteristic	PG			PGOS		
Emulsion capacity (%)	23	±	1a	34.6	±	0.4b
Emulsion stability (%)	6.2	±	0.6a	32.5	±	0.7b
Degree of substitution (%)	0.00	±	0.00a	1.02	±	0.00b
Contact angel (°)	82	±	3a	92	±	3b
Viscosity (cP)	5070	±	14a	5720	±	40b

∗Values indicate mean ± standard deviation for three replicates, while superscript letters indicate significance, *p* < 0.05.

The emulsion capacity and stability of PGOS were higher than that of PG due to the esterification of OSA in PG, resulting in amphiphilic properties in PGOS. The strong thickening capacity of PG is due to the particular structure and properties of PG. The emulsion stability of PG is lower than PGOS because glucomannan is highly hydrophilic (Zhang et al., 2014). Amphiphilic macromolecules can act as effective emulsifiers capable of inhibiting coalescence by adsorbing on the interface and sealing it rapidly to prevent the retraction of newly formed droplets. The emulsifier's structure and the emulsion's viscosity play an essential role in the stabilization of the O/W emulsion (Xu et al., 2015).

**Figure 4:**
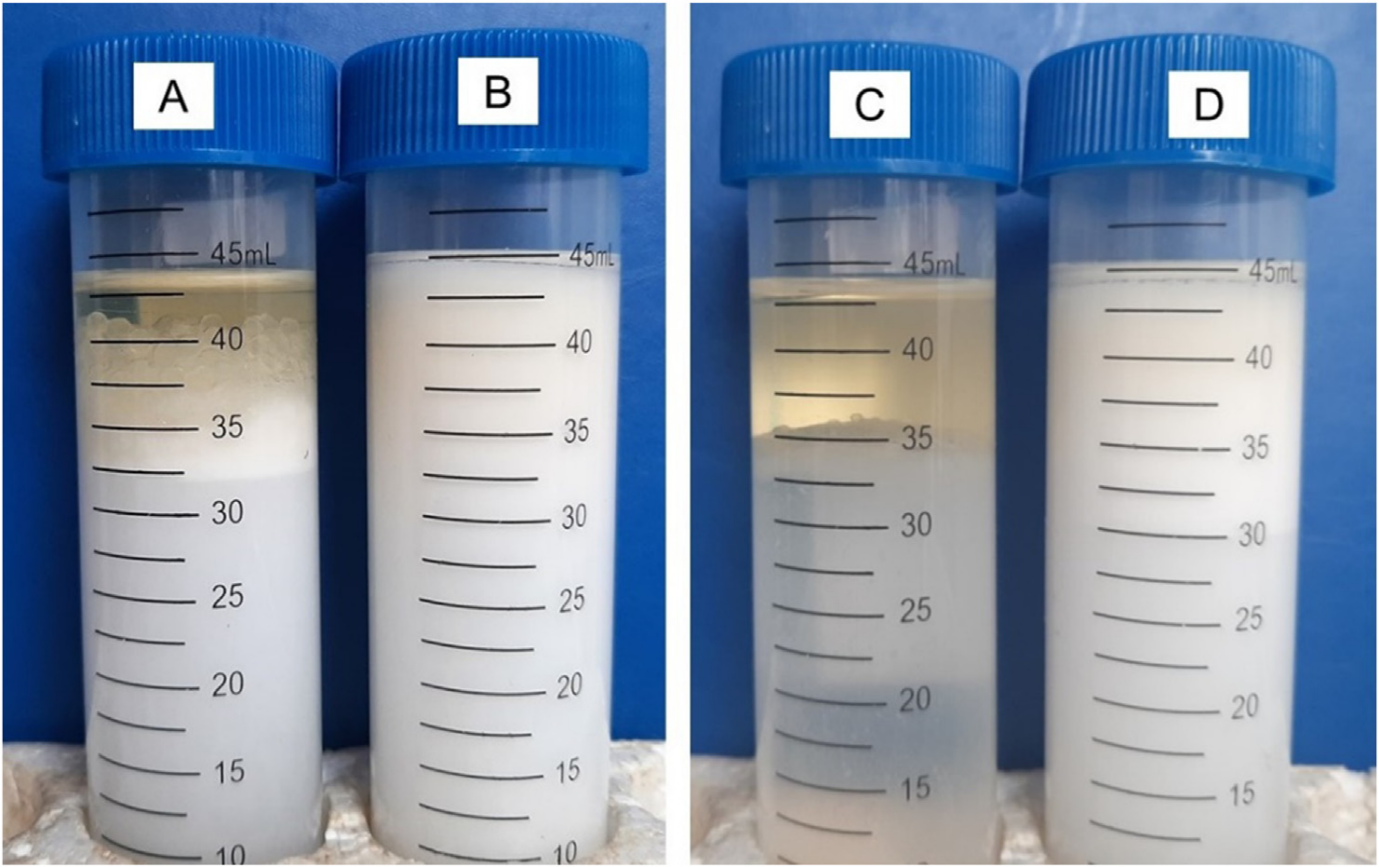
Emulsifying properties of porang glucomannan/PG (A) and octenyl succinic anhydride-modified porang glucomannan/PGOS (B) in oil-water (10 g: 40 g) emulsion. C and D were A and B incubated at room temperature for 30 days, respectively.

Emulsion stability was measured after 30 days of storage at room temperature. Table 4, Figure 4C, and Figure 4D show a decrease in the height of the remaining emulsion layer compared to the initial conditions (Fig. 4A and Fig. 4B). Emulsion capacity decreased from 34.6% to 32.5%. Akbari and Nour (2018) reported that emulsions are thermodynamically unstable and can change over a period of time. Emulsion stability can be affected by storage temperature. Higher storage temperatures can damage the interfacial films formed due to the addition of surfactants. Interfacial films on o/w emulsions can reduce surface tension and increase interfacial viscosity, thereby increasing emulsion stability.

#### 3.3.2 Viscosity

PGOS viscosity is higher than PG viscosity. The PG modification process increased the PGOS viscosity. The increase in viscosity can be caused by the substitution of OSA in PG so that the molecular weight increases. Kazlauske et al. (2017) and Davidovich-Pinhas et al. (2015) reported that the viscosity of ethylcellulose is correlated with its molecular weight. The viscosity increased with the increasing molecular weight of ethyl cellulose. Yanuriati et al. (2017) reported that glucomannan's purity and molecular weight affect its viscosity, where the higher viscosity is caused by an increase in purity and molecular weight. Xu et al. (2015) reported that a large molecular weight of starch-OSA increases the viscosity of the solution, thereby increasing the stability of the emulsion during storage. PGOS showed good emulsion capacity and thickening properties, so that it has very good potential as a surfactant or oleogelator in oleogel production.

#### 3.3.3 Degree of substitution by HPLC

**Figure 5:**
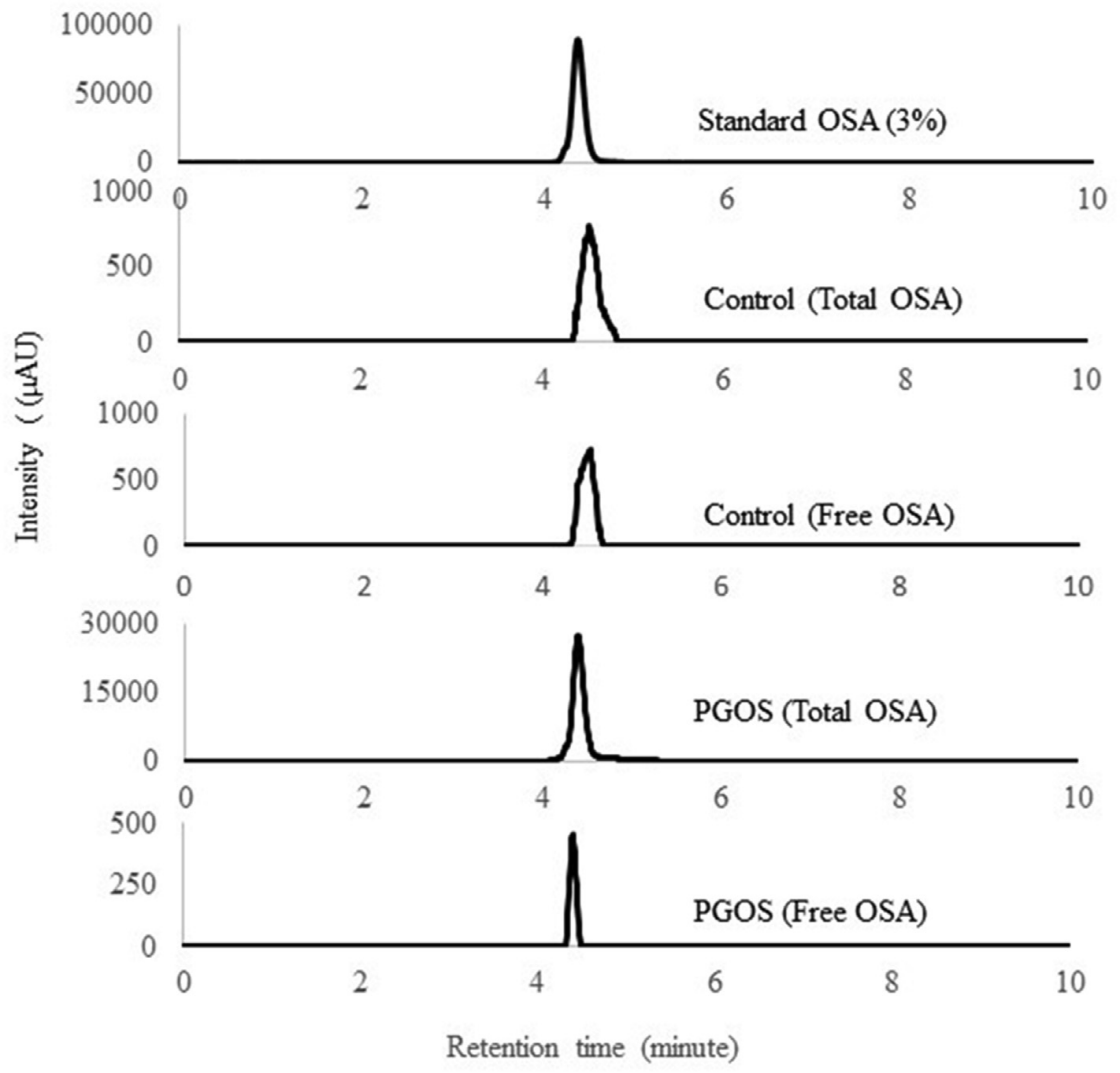
High-performance liquid chromatography/HPLC chromatogram of standard octenyl succinic anhydride/OSA (3%), Control (total and free OSA), OSA modified Porang Glucomannan/PGOS (total and free OSA).

The degree of OSA substitution in PG was analyzed using HPLC. Figure 5 shows that OSA is seen at RT (Retention Time) 4.3 min. The equation obtained based on the standard curve results: Y = 180536x + 99255, R^2^ = 0.997 with a degree of substitution of 1.02%.

The degree of substitution of PG with the addition of OSA was higher than the degree of substitution of waxy rice starch and non-waxy rice starch, which were 0.0262 and 0.0258, respectively (No et al., 2019). The higher degree of substitution can increase emulsifying ability (Mahajan and Sonar, 2019). However, it is lower than Konjac Glucomannan which is 1.51% (Meng et al., 2018). This difference in the degree of substitution is due to differences in porang tuber variety and the purity of extracted glucomannan. Nurlela et al. (2019) reported that glucomannan content was influenced by growth, plant age, climate, growing time, storage, extraction, and purification process.

#### 3.3.4 Hydrophobicity

**Figure 6:**
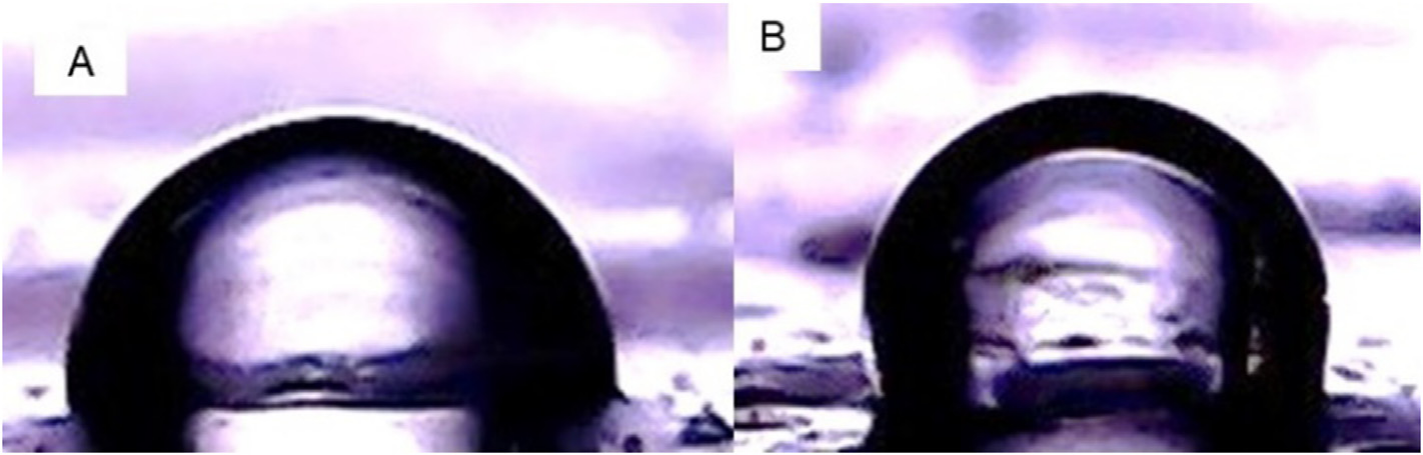
Contact angle porang glucomannan/PG (A) and octenyl succinic anhydride modified porang glucomannan/PGOS (B).

The contact angle is one of the methods used to measure the hydrophobicity of a material. The contact angle is defined as the angle formed between a solid and a liquid. A material surface is called hydrophilic if it has a contact angle of 0≤θ ≤ 90° and material is hydrophobic if it has a contact angle of 90°<θ ≤ 180° (Wahyudi et al., 2019). Table 3 shows that the modification of PG with a combination of Na_2_CO_3_ and OSA can increase the hydrophobicity of PG, which is indicated by increasing the value of the contact angle from 81.5 to 92.5^o^ as shown in Figure 6. Decreasing the polarity on the surface of the films can increase the value of the contact angle (Gutiérrez and González, 2016). Meng et al. (2014) reported that OSA esterification of KGM occurred on the granule surface, which indicated that the surface of the OSA modified KGM granule was smoother than that of the native KGM. This can cause a decrease in the polarity of the films. Meanwhile, Mahajan and Sonar (2019) reported that OSA substitution occurs in the amorphous region of the native starch particles. Starch modification using OSA resulted in decreased moisture sensitivity and increased surface hydrophobicity.

#### 3.3.5 FT-IR analysis

**Figure 7:**
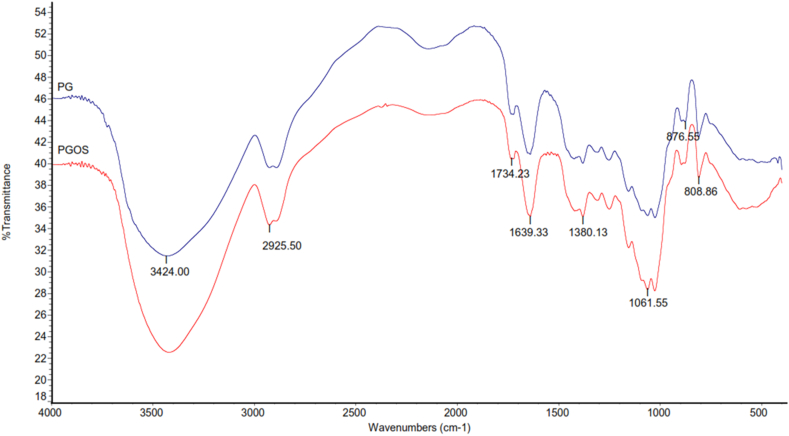
Fourier transform infrared (FT-IR) spectra of the porang glucomannan (PG) and octenyl succinic anhydride-modified porang glucomannan (PGOS).

There are similarities in the characteristics of the functional groups of PG and PGOS based on FT-IR analysis. The FT-IR spectra of the PG and PGOS are shown in Figure 7. The broad peak shows the stretching vibration of the O–H group of methyl in PG at 3424 cm^−1^. The stretching vibrations of the –CH_2_− are shown at ∼2925 cm^−1^. Intrinsic absorption of intramolecular hydrogen bonds is seen at the band of ∼1639 cm^−1^, while the peak of CH appears at 1380 cm^−1^. The stretching of the C=O of the carbonyl of acetyl and OSA groups is shown at ∼1734 cm^−1^ (Manab et al., 2016; Wang et al., 2020; Qiao et al., 2022). However, the intensity of the carbonyl peak in the PGOS spectra was more intense (40.433) than that of the PG (33.400). The increase in the intensity of the PGOS carbonyl peak at 1734 cm^−1^ was due to the esterification of the OSA groups on the PG molecule. Wardhani et al. (2017) reported that the acetyl group in glucomannan could be eliminated by Na_2_CO_3_ (deacetylation), which then the OH groups on deacetylated glucomannan interacts with OSA through an esterification reaction (Meng et al., 2018).

## 4 Conclusion

Optimization of Na_2_CO_3_ and OSA concentrations has resulted in PGOS having amphiphilic properties. The amphiphilic characteristics of PGOS are characterized by increased emulsion capacity and stability as well as hydrophobicity. Degree of substitution, FTIR analysis, and increased viscosity also confirmed that OSA substitution occurred in PG. PGOS can be used as a surfactant or gelator in the production of oleogel.

## Declarations

### Author contribution statement

I Wayan Rai Widarta: Performed the experiments; Analyzed and interpreted the data; Wrote the paper.

Ambar Rukmini: Analyzed and interpreted the data; Contributed reagents, materials, analysis tools or data.

Umar Santoso: Analyzed and interpreted the data; Wrote the paper.

Supriyadi: Contributed reagents, materials, analysis tools or data; Wrote the paper.

Sri Raharjo: Conceived and designed the experiments; Contributed reagents, materials, analysis tools or data; Wrote the paper.

### Funding statement

Dr. Sri – Raharjo was supported by the Ministry of Education, Culture, Research and Technology, Republic of Indonesia [111/E4.1/AK.04. PT/2021].

### Data availability statement

Data included in article/supplementary material/referenced in article.

### Declaration of interests statement

The authors declare no conflict of interest.

### Additional information

No additional information is available for this paper.

## References

[bib1] Akbari S., Nour A.H. (2018). Emulsion types, stability mechanisms and rheology: a review. Int. J. Innov. Res. Sci. Stud..

[bib2] Altuna L., Herrera M.L., Foresti M.L. (2018). Synthesis and characterization of octenyl succinic anhydride modified starches for food applications. A review of recent literature. Food Hydrocolloids.

[bib3] Davidovich-Pinhas M., Barbut S., Marangoni A.G. (2015). The role of surfactants on ethylcellulose oleogel structure and mechanical properties. Carbohydr. Polym..

[bib4] Dermoredjo S.K., Azis M., Saputra Y.H., Susilowati G., Sayaka B. (2021). Sustaining porang (*Amorphophallus muelleri* Blume) production for improving farmers’ income. IOP Conf. Ser. Earth Environ. Sci..

[bib5] Gutiérrez T.J., González G. (2016). Effects of exposure to pulsed light on surface and structural properties of edible films made from cassava and taro starch. Food Bioprocess Technol..

[bib6] Kazlauske J., Gårdebjer S., Almer S., Larsson A. (2017). The importance of the molecular weight of ethyl cellulose on the properties of aqueous-based controlled release coatings. Int. J. Pharm..

[bib7] Li C., Wu K., Su Y., Riffat S.B., Ni X., Jiang F. (2019). Effect of drying temperature on structural and thermomechanical properties of konjac glucomannan-zein blend films. Int. J. Biol. Macromol..

[bib8] Li J., Ye T., Wu X., Chen J., Wang S., Lin L., Li B. (2014). Preparation and characterization of heterogeneous deacetylated konjac glucomannan. Food Hydrocolloids.

[bib9] Mahajan H.S., Sonar Y.A. (2019). Esterification of pea starch with octenyl succinic anhydride using conventional and microwave irradiation method: synthesis and characterization. J. Polym. Res..

[bib10] Manab A., Purnomo H., Bambang Widjanarko S., Eka Radiati L. (2016). Modification of porang (Amorphophallus oncophyllus) flour by acid and thermal process using conventional heating in waterbath and microwave irradiation. Adv. J. Food Sci. Technol..

[bib11] Meng F., Zheng L., Wang Y., Liang Y., Zhong G. (2014). Preparation and properties of konjac glucomannan octenyl succinate modified by microwave method. Food Hydrocolloids.

[bib12] Meng F.B., Li Y.C., Liu D.Y., Zhong G., Guo X.Q. (2018). The characteristics of konjac glucomannan octenyl succinate (KGOS) prepared with different substitution rates. Carbohydr. Polym..

[bib13] No J., Mun S., Shin M. (2019).

[bib14] Nurlela, Ariesta N., Santosa E., Muhandri T. (2019). Effect of harvest timing and length of storage time on glucomannan content in porang tubers. IOP Conf. Ser. Earth Environ. Sci..

[bib15] Qiao D., Lu J., Shi W., Li H., Zhang L., Jiang F., Zhang B. (2022). Deacetylation enhances the properties of konjac glucomannan/agar composites. Carbohydr. Polym..

[bib16] Sadhukhan B., Mondal N.K., Chattoraj S. (2016). Optimisation using central composite design (CCD) and the desirability function for sorption of methylene blue from aqueous solution onto Lemna major. Karbala Int. J. Mod. Sci..

[bib17] Shi Y., Li C., Zhang L., Huang T., Ma D., Tu Z. cai, Wang H., Xie H., Zhang N. hai, Ouyang B. ling. (2016). Characterization and emulsifying properties of octenyl succinate anhydride modified Acacia seyal gum (gum Arabic). Food Hydrocolloids.

[bib18] Soto-Muñoz L., Palou L., Argente-Sanchis M., Ramos-López M.A., Pérez-Gago M.B. (2021). Optimization of antifungal edible pregelatinized potato starch-based coating formulations by response surface methodology to extend postharvest life of ‘Orri’ mandarins. Sci. Hortic. (Amst.).

[bib19] Sun Z., Yan X., Xiao Y., Hu L., Eggersdorfer M., Chen D., Yang Z., Weitz D.A. (2022).

[bib20] Wahyudi, Subagyo R., Gapsari F. (2019). Physical and chemical mechanisms of hydrophobicity of nanoparticle membranes (Mg+Al2O3). J. Achiev. Mater. Manuf. Eng..

[bib21] Wang Y., Liu J., Li Q., Wang Y., Wang C. (2015). Two natural glucomannan polymers, from Konjac and Bletilla, as bioactive materials for pharmaceutical applications. Biotechnol. Lett..

[bib31] Wang B., Tian H., Xiang D. (2020). Stabilizing the oil-in-water emulsions using the mixtures of **Dendrobium officinale** polysaccharides and gum Arabic or propylene glycol alginate. Molecules.

[bib22] Wardhani D.H., Puspitosari D., Ashidiq M.A., Aryanti N., Prasetyaningrum A. (2017). Effect of deacetylation on functional properties of glucomannan. AIP Conf. Proc..

[bib23] Xu Y., Huang Q., Fu X., Jane J. lin. (2015). Modification of starch octenylsuccinate by β-amylase hydrolysis in order to increase its emulsification properties. Food Hydrocolloids.

[bib24] Yang D., Yuan Y., Wang L., Wang X., Mu R., Pang J., Xiao J., Zheng Y. (2017). A review on konjac glucomannan gels: microstructure and application. Int. J. Mol. Sci..

[bib25] Yanuriati A., Marseno D.W., Rochmadi, Harmayani E. (2017). Characteristics of glucomannan isolated from fresh tuber of Porang (Amorphophallus muelleri Blume). Carbohydr. Polym..

[bib26] Ye X., Li P., Lo Y.M., Fu H., Cao Y. (2019). Development of novel shortenings structured by ethylcellulose oleogels. J. Food Sci..

[bib27] Zainal Abiddin N.F., Yusoff A., Ahmad N. (2015). Optimisation of reaction conditions of octenyl succinic anhydride (OSA) modified sago starch using response surface methodology (RSM). Int. Food Res. J..

[bib28] Zhang C., Chen J. Da, Yang F.Q. (2014). Konjac glucomannan, a promising polysaccharide for OCDDS. Carbohydr. Polym..

[bib29] Zhao S., Tian G., Zhao C., Lu C., Bao Y., Liu X., Zheng J. (2018). Emulsifying stability properties of octenyl succinic anhydride (OSA) modified waxy starches with different molecular structures. Food Hydrocolloids.

[bib30] Zhong G., Meng F.B., Li Y.C., Liu D.Y., Guo X.Q., Zheng L.J. (2018). Structure and rheological characterization of konjac glucomannan octenyl succinate (KGOS). Food Hydrocolloids.

